# Facile Precursor for Synthesis of Silver Nanoparticles Using Alkali Treated Maize Starch

**DOI:** 10.1155/2014/702396

**Published:** 2014-10-29

**Authors:** M. H. El-Rafie, Hanan B. Ahmed, M. K. Zahran

**Affiliations:** ^1^Textile Research Division, National Research Centre, Dokki, Cairo 12311, Egypt; ^2^Chemistry Department, Faculty of Science, Helwan University, Ain Helwan, Cairo 11795, Egypt

## Abstract

Silver nanoparticles were prepared by using alkali treated maize starch which plays a dual role as reducer for AgNO_3_ and stabilizer for the produced AgNPs. The redox reaction which takes a place between AgNO_3_ and alkali treated starch was followed up and controlled in order to obtain spherical shaped silver nanoparticles with mean size 4–6 nm. The redox potentials confirmed the principle role of alkali treatment in increasing the reducibility of starch macromolecules. The measurements of reducing sugars at the end of reaction using dinitrosalicylic acid reagent (DNS) were carried out in order to control the chemical reduction reaction. The UV/Vis spectra show that an absorption peak, occurring due to surface plasmon resonance (SPR), exists at 410 nm, which is characteristic to yellow color of silver nanoparticles solution. The samples have been characterized by transmission electron microscopy (TEM), which reveal the nanonature of the particles.

## 1. Introduction

Noble metal nanoparticles (NPs) are envisaged to provide solutions to optical, electronic, biotechnological, and environmental challenges in the areas of solar energy conversion, catalysis, medicine, and water treatment [1]. Kamat also confirmed that size, shape, and surface morphology play pivotal roles in controlling the physical, chemical, optical, and electronic properties of these nanoscopic materials [2]. When macroscaled counterparts of nanometals are compared with that of metal ions, they often show unique and considerably changed physical, chemical, and biological properties [3].

Also a fact has been established that the size, morphology, stability, and physicochemical properties of the metal NPs are strongly influenced by the experimental conditions, the kinetics of interaction of metal ions with reducing agents, and adsorption processes of stabilizing agent with metal NPs [4, 5]. Thus, the synthesis of noble metal NPs for various novel applications has become a major field of research interest [6].

Colloidal solutions of silver nanometals have been particularly studied because of their characteristic properties, such as catalytic ability, antibacterial activity, good conductivity, and chemical stability [7]. Chemical reduction by the colloidal route is the most frequently applied method for the preparation of silver nanoparticles (AgNPs) due to the stable colloidal dispersions in water or in organic solvents [8, 9] and via microemulsion [10], polymer protection methods [11, 12], carbon nanotube [13], coprecipitation [13, 14], liquid crystals [15], biological macromolecules [16, 17], latex particles [18], dendrimers [19–21], microgels, and hydrogels [22, 23].

Also some commonly chemical reductants are used for preparation of nanosilver like borohydride, ascorbate, hydrazine, and elemental hydrogen [24–30].

Schneider et al. indicated that using of a strong reductant such as borohydride resulted in small particles that were somewhat monodispersed, but the generation of larger particles was difficult to control. However, using of a weaker reductant such as citrate, resulted in a slower reduction rate [8, 9]. It could be suggested that the high surface energy of these particles may make them extremely reactive, and most systems undergo aggregation without protection or passivation of their surfaces [31–35].

Thus, it could be confirmed that NPs synthesis not only requires a reductant, but also requires the presence of stabilizer. Some of the commonly used methods for surface passivation include protection by self-assembled monolayers, the most popular being citrate and thiol-functionalized organics [32], encapsulation in the H_2_O pools of reverse microemulsions [36], and dispersion in polymeric matrixes [37–39]. Biological applications of nanometals such as in biosensors [40] and molecular labeling [41] require the use of biocompatible materials for synthesis and stabilization.

The chemical methods for synthesis of nanomaterials using organic solvents and toxic reducing agents have some disadvantages, including low yield, high-energy requirements, and a need for difficult and wasteful purifications [37, 42]. Thus, it is essential to develop efficient green synthetic methods [38, 43]. Some of the previously developed green methods have reported the use of reagents, such as biological materials, Tollens' reagent, and techniques such as irradiation for synthesizing NPs in green methods [44].

Previous researches detailed with reduction and stabilization of metal NPs by natural polymers like starch [37, 45, 46], cellulosic products [47, 48], carboxymethyl cellulose [38], chitosan [49, 50], and gum acacia [51–53], alginate [54], and pectin [55] have been studied.

Among natural polymers, the use of starch is applied in different fields as it is one of the most promising biocompatible and biodegradable materials that is universally available and of low cost. Starch as a widely available raw material seems to be a very good substrate for preparation of nanoparticles [37, 56, 57]. Starch is a linear polymer (polysaccharide) made up of repeating glucose units linked by *α*1-4 glucosidic linkages. There are two major molecular components in starch: 10–30% linear amylose and 70–90% branched amylopectin [58]. They are organized in alternating crystalline and amorphous lamellae in the granules [59, 60].

Starches are used in large quantities in various industrial applications. They are used to provide body and consistency to solutions, as a vehicle for transferring colors, as adhesives for paper and paper products, as a sizing agent in textiles, and as a source of energy in human and animal diets. However, natural starches often do not match the properties required for a particular end-use. Many starches show unstable viscosity when their pastes are subjected to high shearing action, heated for prolonged periods, or subjected to freeze-thaw cycles. There is thus the necessity of modifying the starches. [37].

The objective of this study is focusing on a green synthesis of AgNPs via investigating and following up the ability of alkali treated maize starch to play the dual role of reductant to the silver nitrate (generator of AgNPs) and stabilizer for the produced nanoparticles without using any harazad chemicals or any intermediate complicated steps.

## 2. Experimental

### 2.1. Materials and Chemicals

Silver nitrate (99.5%), maize starch supplied from Egyptian Starch and Glucose Company, Cairo, Egypt, sodium hydroxide, 3,5-dinitro salicylic acid (DNS), sodium sulphite, potassium sodium tartrate, glucose, phenol, and sodium carbonate monohydrate were all used as received.

### 2.2. Method

Different weights of native maize starch (1–6 g/L) were treated with sodium hydroxide solution (20 g/L) using magnetic stirrer to prepare different solutions of alkali treated maize starch with pH value nearly 12 in order to serve the dual role as a reductant and stabilizer for the preparation of nanosilver. After complete dissolution the temperature of the reaction medium was raised to the desired degree (50–80°C). In this moment, certain amount from silver nitrate solution (0.1 mole/L) was added dropwise (keeping in mind that the total volume of the reactants is 100 mL). The reaction was kept under continuous stirring for different durations (15–90 minutes). After addition of silver nitrate, the reaction medium acquires a yellowish color indicating the formation of silver nanoparticles. The progression of the reaction was controlled by UV-visible absorption; aliquots from the reaction bulk were withdrawn at given time intervals and evaluated.

## 3. Measurements

### 3.1. Measurement of Redox Potentials

The redox potential of different reducing sugars (glucose, fructose, and starch), at certain concentration (1 g/L), with sodium hydroxide (20 g/L), was measured over a range of temperatures from 60 to 80°C. The reducing sugar was dissolved in 100 cm^3^ of distilled water before sodium hydroxide was added. After dissolution, the pH was recorded, the solution was heated with stirring till reaching the desired degree, and then sodium hydroxide is added. Redox potential was measured using an oxidation-reduction potential platinum electrode connected to a pH meter and recorded in −mV.

### 3.2. Determination of the Concentration of Reducing Sugars

The dinitrosalicylic acid reagent (DNS) was used for the determination of concentrations of reducing sugars. This method tests for the presence of the free carbonyl group of the so-called reducing sugars remaining after the redox reaction between silver nitrate and alkali treated starch. This involves the oxidation of the aldehydic and/or ketonic groups present in the sugar to the carboxylic group and 3,5-dinitrosalicylic acid is reduced to 3,5-diaminosalicylic acid under alkaline conditions.

The reagent is composed of dinitrosalicylic acid, Rochelle salt (sodium potassium tartrate), phenol, sodium bisulfite, and sodium hydroxide. According to Sumner [61], Rochelle salt is added to prevent the reagent from dissolving oxygen, phenol is used to increase the amount of color produced, and bisulfite is used to stabilize the color obtained in the presence of phenol. Also the alkali is required for catalyzing the reducing action of sugar on dinitrosalicylic acid.

The test was carried out with 3 mL of DNS reagent which is added to 3 mL of different colloidal silver nanoparticles solutions under different conditions in capped test tubes. The mixture was heated at 90°C for 15 minutes to develop the reddish brown color. 1 mL of Rochelle salt is added to stabilize the color. After cooling at room temperature in a cold water bath, the absorbance was recorded spectrophotometrically at 575 nm [61].

### 3.3. UV-Visible Spectroscopy

Silver nanoparticles solutions exhibit an intense absorption peak due to the surface plasmon resonance (SPR). Thus the UV-visible absorption spectra were used to prove the formation of AgNPs colloidal solutions. The UV-visible absorption spectra of AgNPs colloidal solutions were measured using a multichannel spectrophotometer (T80 UV/VIS, *d* = 10 mm, PG Instruments Ltd, Japan) at wavelengths 250–600 nm.

### 3.4. Transmission Electron Microscope (TEM)

For more characterization of the prepared silver nanoparticles, two drops of the silver nanoparticles colloidal solutions were placed on a 400 mesh copper grid coated by carbon film and then the grids was conducted to the morphology and the distribution of AgNPs were characterized by means of a JEOL-JEM-1200 transmission electron microscope.

### 3.5. Particles Size Distribution

The diameter and distribution of silver nanoparticles in solution were calculated by 4 pi analysis software using TEM photos. The average diameter of the silver nanoparticles was determined from the diameter of at least 20–100 nanoparticles found in several chosen areas in enlarged microphotographs.

## 4. Results and Discussion

### 4.1. Mechanism for Synthesis of AgNPs Using Alkali Treated Maize Starch

According to the studies reported by Bankura on the preparation of AgNPs using alkali treated dextran as an example of naturally occurring polysaccharide, the method for the preparation of silver nanoparticles using alkali treated starch as both reducing and protecting agent in water could be quite simply suggested as follows: immediate gelatinization of starch in sodium hydroxide solutions suggests that the presence of sodium hydroxide increases the affinity of starch granules to water and makes them birefringence; as a consequence swelling of starch granules occurs very fast and results in significant increment in granules size. Thus, alkali treatment is supposed to increase the solubility of starch by rupturing the granules and then degrading starch macromolecules to give smaller fragments with higher reducing power; also it acts in increasing the affinity of the free hydroxyl groups of sugars by removing the protons which in turns assist the formation of silver nanoparticles.

For synthesis of AgNPs, the generally accepted mechanism suggests a two-step process, that is, atom formation and then polymerization of the atoms. In the first step, a portion of metal ions in a solution is reduced by the reducing agent (starch fragments). In the second step, the atoms produced act as nucleation centers and catalyze the reduction of the remaining metal ions present in the bulk solution. Subsequently, the atoms coalesce leading to the formation of metal clusters. Since the binding energy between two metal atoms dimerize or associate with excess ions, the surface ions again reduce and in this way the aggregation process does not cease until high values of nuclearity are attained, which results in larger particles. The process is stabilized by the interaction with the polymer so preventing further coalescence and aggregation [62].

### 4.2. Monitoring of Redox Potentials for Various Reducing Sugars

Oxidation-reduction potential (ORP or redox potential) measures an aqueous system's capacity to either release or accept electrons from chemical reactions. When a system tends to accept electrons, it is an oxidizing system. When it tends to release electrons, it is a reducing system. A system's reduction potential may change upon introduction of a new species or when the concentration of an existing species changes. Since the ORP potential is temperature dependent, temperature must also be recorded with time in order to permit comparison of the different ORP values.

The redox potentials of alkali treated glucose and fructose as monosaccharides and alkali treated starch macromolecules were recorded and compared.

**Figure 1:**
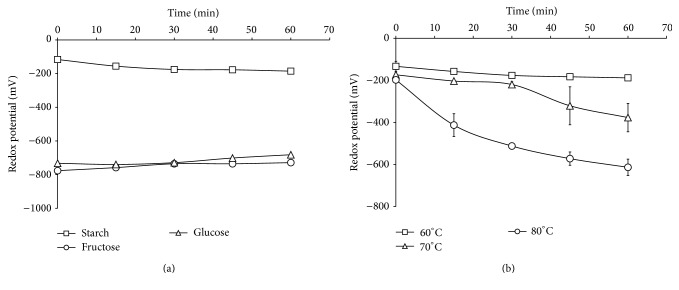
(a) Redox potentials for glucose and fructose as monosaccharides compared with starch as a polysaccharide using 1 g/L of sugar, 20 g/L NaOH at 60°C. (b) Redox potentials for starch at different reaction temperatures using 1 g/L starch, 20 g/L NaOH.

From Figure 1(a), it was observed that different sugars at equal concentrations gave different redox potentials. Also, it was clear from the redox potentials of the two monosaccharides studied that their reducing power was close to each other, and it could be explained as follows: although D-fructose would appear to be nonreducing, it readily undergoes keto-enol tautomerism at high pH to form a mixture of D-glucose and D-mannose.

In addition, treatment of D-fructose with alkali may even cause decomposition of the carbon chain to form more products with reducing capability [63]; hence the reducing potential is observed. Thus, in case of starch, it may be supposed that the alkali treatment acts in decomposition of starch polymeric chains to generate fragments with higher reducibility, which, in turn, acts as excellent reducers of AgNO_3_ for the preparation of silver nanoparticles.

Figure 1(b) shows the redox potentials of alkali treated starch solutions prepared at different temperatures (60, 70, and 80°C) with time. It is clear from the data that the temperature plays an important role in reduction reaction, as by raising the reaction temperature from 60 up to 80°C, the redox potentials are increased, which could be attributed to the important role of temperature in accelerating the reduction reaction and producing greater amounts of reducing fragments in shorter time, and so higher redox potential values are observed.

### 4.3. Follow-Up of Redox Reaction between AgNO_3_ and Alkali Treated Starch: Determination of Residual Reducing Sugars Using Dinitrosalicylic Acid Reagent

All the simple sugars (monosaccharide) and maltose are reducing sugars, whereas sucrose (table sugar) and starch are nonreducing carbohydrates. The reducing sugars have free aldehyde or keto groups. Under alkaline and heating conditions, they can react with DNS to produce 3-amino-5-nitrosalicylate (red brown), which has a maximum absorbance at 575 nm, while the reducing sugars are oxidized into gluconate and other products. Over a certain range of concentrations, the amount of reducing sugars is proportional to the depth of the color. By making a standard curve, the content of reducing sugars and total sugar (nonreducing sugars are hydrolyzed into reducing sugars) can be determined spectrophotometrically.

Alkali treatment of maize starch was carried out in the current work in order to increase the solubility and provide more fragments with higher affinity and reducibility which are required for synthesis of maximum concentrations of stable colloidal silver nanoparticles solutions.

Maize starch macromolecules consist of linear polymeric polysaccharide (amylose) and highly branched polysaccharide (amylopectin). With alkali treatment of starch, granules are supposed to rupture giving more fragments with higher reducing capability, which in turn increases the swellability, solubility, and then the accessibility.

This method [64] tests for the presence of free carbonyl groups, of reducing sugars produced from starch fragmentation, as during the redox reaction between sugars (starch fragments) and silver nitrate, the sugars are turned to the oxidized form with more aldehydic and/or ketonic groups. However, this method involves the oxidation of the aldehydic and/or ketonic groups of sugars presenting in the reaction medium to the corresponding carboxylic acid, and simultaneously, 3,5-dinitrosalicylic acid is reduced to 3,5-diaminosalicylic acid. This method depends upon the fact that all higher oligosaccharides starting with maltose would produce equivalent amounts in color with DNS reagent; that is, increasing of color intensity means higher concentrations of starch fragments.

Thus it could be concluded that increasing the concentration of reducing sugars equalizes and conforms the redox reaction between starch and silver ions and so means increasing the affinity for building of silver clusters.

### 4.4. Effect of Starch Concentration

**Figure 2:**
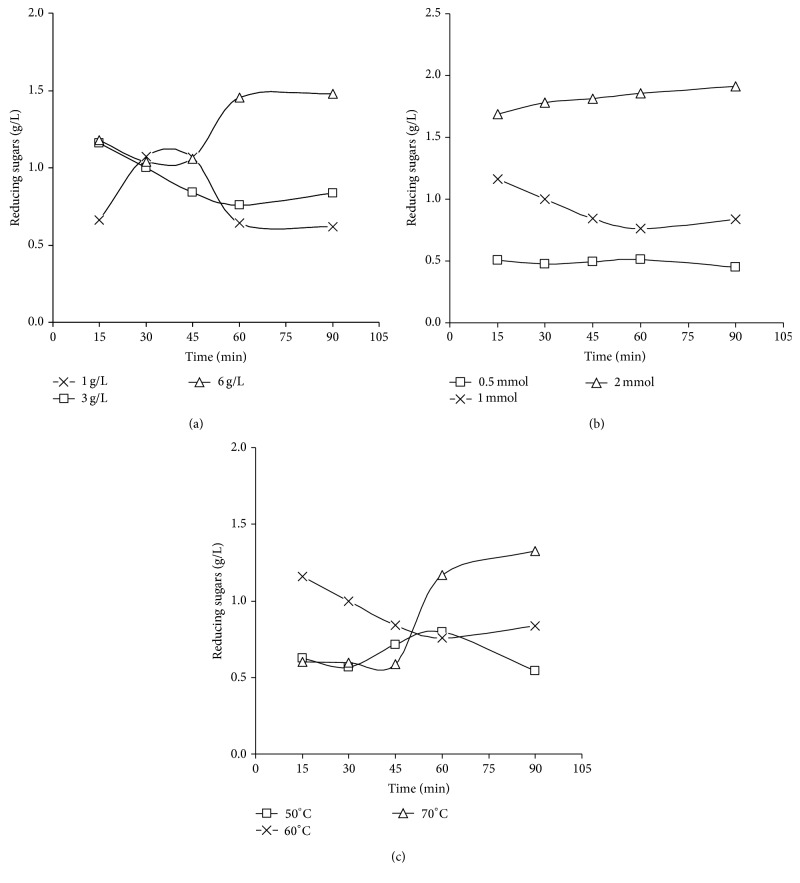
(a) Reducing sugar content as a function of starch concentration using 20 g/L NaOH and 1 mmole AgNO_3_ at 60°C. (b) Reducing sugar content as function of silver nitrate concentration with reaction time by using 1 g/L maize starch and 20 g/L NaOH at 60°C. (c) Reducing sugar content as function of temperature with reaction time by using 1 g/L maize starch, 1 mmole AgNO_3_, and 20 g/L NaOH.

From data shown in Figure 2(a), it could be supposed that increasing the concentration of starch in the reaction medium caused an enhancement of reduction process by time, so this means that the starch fragmentation is proceeded giving higher amounts of reducing sugars, which, in turn, are required for silver nanoparticles preparation [54, 55]. Also the alkali treated starch solution without silver nitrate was monitored with time and the concentration of reducing sugars was not changed significantly (0.149 ± 0.009 g/L), confirming that reducing sugars resulted as products of the redox reaction which takes place between starch fragments and silver nitrate.

### 4.5. Effect of Silver Nitrate Concentration and Temperature

From Figure 2(b), it could be clarified that when the concentration of silver nitrate was increased, the concentration of reducing sugars was raised, as it approached the maximum value (1.6 g/L) with the highest concentration of silver nitrate (2 mmole/L), as by increasing the concentration of silver nitrate, the rate of redox reaction is increased, resulting in higher concentration of reducing sugars [54, 55].

From Figure 2(c), by time, the maximum concentration of reducing sugars (1.3 g/L) was approached at 70°C. So, it could be decided that higher temperature plays an important role in accelerating the redox reaction between silver ions and starch macromolecules (as suggested by redox potential data).

So, it could be concluded that this test may help in following up the redox reaction between silver ions and alkali treated starch, for controlling the reaction to achieve the optimum conditions for preparation of smallest sized AgNPs.

### 4.6. UV-Vis Spectroscopic Analysis

**Figure 3:**
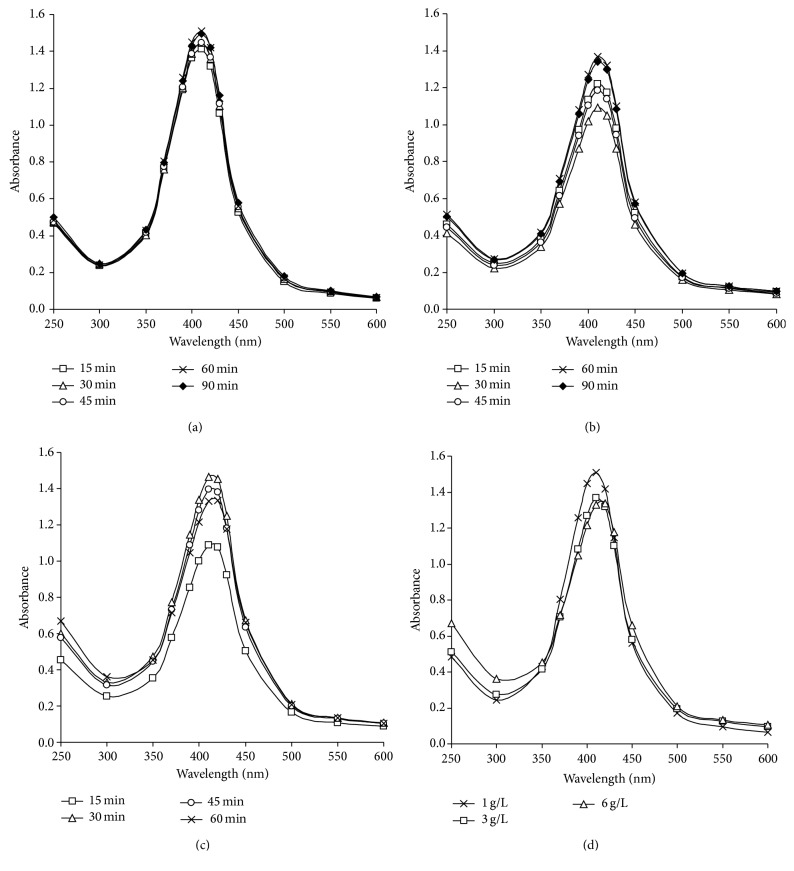
UV-Vis absorbance of silver nanoparticles solutions by time, using 1 mmole/L AgNO_3 _with 20 g/L NaOH at 60°C. (a) 1 g/L starch, (b) 3 g/L starch, and (c) 6 g/L starch. (d) UV-Vis absorbance of silver nanoparticles solutions by using different concentrations of maize starch, using 1 mmole/L AgNO_3 _with 20 g/L NaOH at 60°C for 60 minutes.

**Figure 4:**
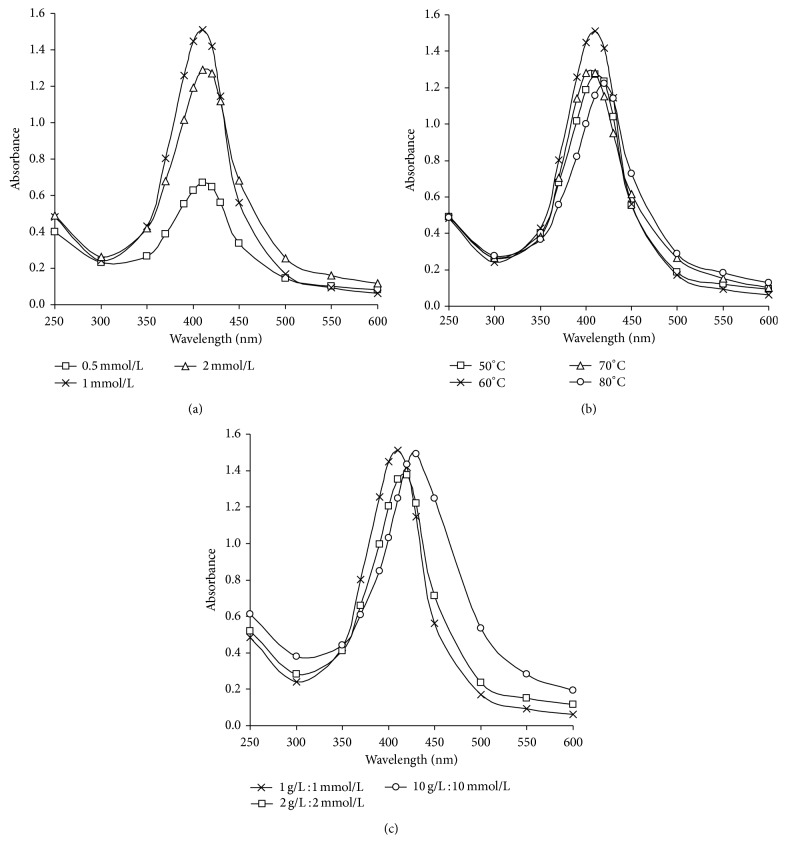
(a) UV-Vis absorbance for AgNPs colloidal solutions by using different concentrations of AgNO_3_ (0.5–2 mmole/L), with 1 g/L starch and 20 g/L NaOH at 60°C for 60 minutes. (b) UV-Vis absorbance for AgNPs colloidal solutions at different temperatures, using 1 g/L of starch with 20 g/L NaOH, 1 mmole/L AgNO_3_. (c) UV-Vis absorbance for AgNPs colloidal solutions at different ratios between starch and AgNO_3_; the experiments are carried out at 60°C for 60 minutes.

Silver nanoparticles absorb radiation in the visible region of the electromagnetic spectrum (380–450 nm) due to the surface plasmon resonance (SPR) transition. This SPR transition is responsible for the striking yellowish brown coloration of silver nanoparticles. The UV-Vis absorption spectrum of the silver nanoparticles is shown and discussed in Figures 3 and 4. The absorbance of the silver nanoparticles is observed at 410 nm. It must be noted that all samples under different experimental conditions are diluted before measuring process till the concentration of the produced nanosilver reaches 10 ppm.

From Figure 3 which shows the UV-Vis spectra of the colloidal solutions of nanosilver prepared by different concentrations of maize starch, it could be clarified that, from Figures 3(a) and 3(b) showing the spectra of 1 g/L and 3 g/L of maize starch, the absorbance was increased with time till it reached the maximum value at 60 minutes (1.5 at 410 nm for 1 g/L and 1.3 at 415 nm for 3 g/L resp.) and further increase in the reaction duration leads to decreasing in absorbance; however, in Figure 3(c) showing the spectral data of 6 g/L maize starch, the absorbance value increased with time till it reached maximum value after 30 minutes (1.46 at 415 nm) and started to decrease.

These findings could be attributed to the fact that prolonging the reaction duration for samples prepared at 60°C leads to marginal increase in the plasmon intensity indicating that silver ions are reduced and are used for cluster formation; however, by time, decreasing of absorbance values and shifting to longer wavelength may be related to the aggregation of the prepared nanoparticles.

Figure 3(d) shows the comparable UV-Vis spectra of the silver colloidal solutions obtained using different concentrations of maize starch as reducing and stabilizing agent. The results bring into focus a number of observations which may be summarized as follows: maximum intensity of the plasmon peak (410 nm) is observed by using 1 g/L of maize starch at pH 12 which indicates a full reduction of silver ions, therefore reflecting the dual role of starch as reducing and efficient stabilizing agent in alkaline medium.

The maximum peak kept at 410 nm although the absorbance value decreases by increasing the concentration of starch; it could be explained as follows: the silver particle size does not change significantly [65], and almost no agglomeration of silver nanoparticles could be supposed (Figure 3(d)). This phenomenon reveals that the silver nanoparticles are well capped with starch macromolecules.

Taking inmind the concentration of the formed reducing sugars during the reaction (Figure 2(a)), by increasing the reaction duration, it could be concluded that the amount of reducing sugars is increased by time till 60 minutes with increasing the concentration of starch in the reaction medium from 3 g/L to 6 g/L. So, the increment in reduction is accompanied by decrement in the role of alkali treated starch as stabilizing agent, which is confirmed by the results obtained in Figure 3(d).

### 4.7. Effect of Silver Nitrate Concentration

From the data shown in Figure 4(a), it is clear that the maximum peak kept at 410 nm at different concentrations of silver nitrate, and this clarifies the efficiency of alkali treated starch in acting the dual role as reducer and stabilizer for silver nanoparticles. Maximum absorbance value (1.5 at 410 nm) is recorded by using 1 mmole/L silver nitrate to prepare 108 ppm silver nanoparticles at 60 minutes; however further increase in the concentration of silver nitrate (2 mmole/L) leads to decrease in the absorbance value and shifting of plasmon peak to longer wavelength (1.3 at 415 nm), which may be attributed to aggregation and agglomeration of the produced nanoparticles.

### 4.8. Effect of Temperature

Figure 4(b) shows the UV-Vis absorption spectroscopy of 108 ppm AgNPs prepared by 1 g/L maize starch, 20 g/L NaOH, and 1 mmole/L silver nitrate at different temperatures (50, 60, 70, and 80°C) using initial pH 12 for 60 min. It is clear from the data that (a) the temperature plays an important role in both reduction reaction and particle size, (b) when the reaction temperature was 50°C the color of the solution was light yellow, and the intensity of the plasmon band was little more broaden around 410 nm which indicates lower conversion percentage of Ag^+^ to Ag^0^, (c) raising the reaction temperature up to 60°C is accompanied by formation of deep yellow color and the absorption band at 410 nm becomes stronger and narrower which means higher conversion of Ag^+^ to Ag^0^ with smaller nanoparticles size, and (d) further increases in the reaction temperature up to 70 and 80°C lead to lower absorption intensity and shifted towards longer wavelength band (415 nm), which could be attributed to those silver nanoparticles which could be formed with enlarged size, may be collapsed together, and starts to agglomerate.

So, it could be concluded that the desired conditions for preparing 108 ppm silver nanoparticles using maize starch are 1 g/L starch, 20 g/L NaOH, and 1 mmole/L silver nitrate at 60°C for 60 minutes.

The disadvantage of preparing AgNPs by increasing concentration of silver nitrate (Figure 4(a)) and by raising the reaction temperature (Figure 4(b)) could be related to the increment in the formed reducing sugar in the reaction medium (Figures 2(b) and 2(c)) and thereby decrement in the stabilizing efficiency of the formed AgNPs.

### 4.9. Preparation of Silver Nanoparticles Colloidal Solutions for Industrial Applications

In order to achieve efficient reduction and best stability for conversion silver ions to silver nanosized metallic form, certain ratio of silver nitrate to alkali treated starch must be established. Hence, preparation of silver nanoparticles was carried out using higher concentrations of both silver nitrate and starch. Increasing the concentration of silver nitrate and starch was done while the ratio of silver nitrate to starch is maintained.

Figure 4(c) shows the UV-Vis spectra of the silver colloidal solutions obtained using different ratios of alkali treated maize starch and silver nitrate. It could be significantly observed that by increasing the ratio from 1 g/L starch : 1 mmole/L AgNO_3_ (for preparing 108 ppm AgNPs) up to 2 g/L starch : 2 mmole/L AgNO_3_ (for preparing 216 ppm AgNPs), the maximum absorption peak at 410 nm becomes stronger and narrower which indicates that silver nanoparticles are formed in higher amounts with small size; however, further increment of ratio up to 10 g/L starch : 10 mmole/L AgNO_3_ (for producing 1080 ppm AgNPs) leads to higher absorption intensity shifted to longer wavelength which may be explained by the formation of geometrically different silver nanoparticles.

Thus, it could be suggested that the desired conditions for preparing 1080 ppm AgNPs using alkali treated maize starch is 10 g/L maize starch, 20 g/L NaOH, and 10 mmole/L AgNO_3_, at 60°C for 60 minutes.

### 4.10. TEM Graphs

**Figure 5:**
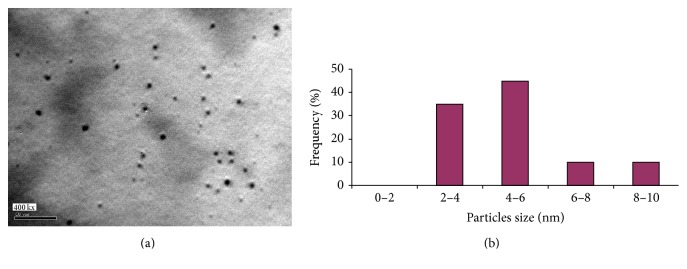
(a) TEM micrograph of silver nanoparticles prepared with 1 g/L maize starch and 1 mmole/L AgNO_3_ at pH 12 and 60°C for 60 minutes. (b) Size particles distribution and size average for silver nanoparticles.

**Figure 6:**
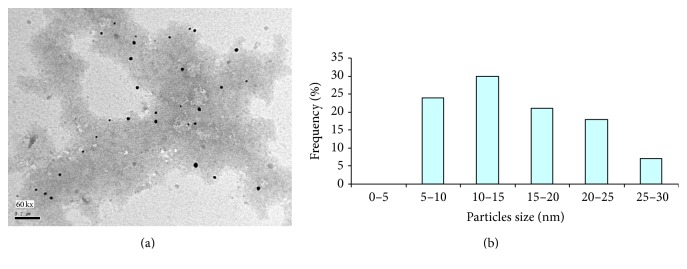
(a) TEM micrograph of silver nanoparticles prepared with 3 g/L maize starch using 1 mmole/L AgNO_3_ at pH 12 and 60°C for 60 minutes. (b) Size distribution and size average for silver nanoparticles.

**Figure 7:**
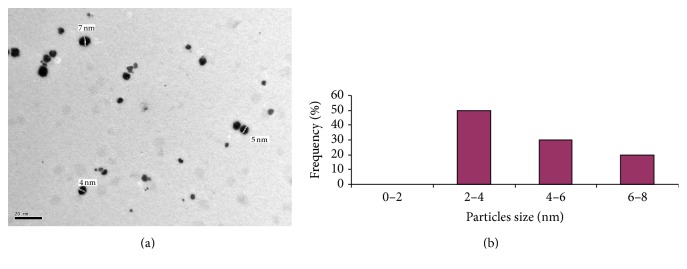
(a) TEM micrograph of silver nanoparticles prepared with 10 mmole/L silver nitrate and 10 g/L maize starch at pH 12 and 60°C for 60 minutes. (b) Size distribution and size average for silver nanoparticles.

To confirm the results obtained from the UV-Vis absorption spectroscopy, the size determination and size distribution of AgNPs were further established by detecting TEM photos of silver colloids prepared by alkali treated starch as reducing agent for silver nitrate and stabilizing agent for the produced nanoparticles. From Figures 5(a), 6(a), and 7(a), the spherical shaped silver nanoparticles were seen. Size distribution and size average are presented in Figures 5(b), 6(b), and 7(b).

From the data shown, it could be concluded that size distribution of AgNPs for all samples prepared was 2–30 nm. By using 1 g/L starch, size distribution of AgNPs was in range of 2–10 nm (Figure 5(a)). The maximum size average of AgNPs was 4–6 nm (ca. 40%) as shown in Figure 5(b). From Figure 6(a) size distribution of silver nanoparticles prepared with 3 g/L starch (Figure 6(b)); little enlarged nanosized spherical particles are formed with 3 g/l starch compared with 1 g/L starch. Also the histogram (Figure 6(b)) clearly illustrates that the average prepared particle size seemed to be around 10–15 nm (ca. 30%).

Obviously, then, the concentration of starch plays an important role in the reduction and stabilization process involved in the preparation of nanoparticles, as the particle size decreases significantly with decreasing the concentration of starch, a point which implies that 1 g/L is enough concentration of starch in order to get the best dispersibility, and so it can form perfect protection layer for silver nanoparticles to prevent agglomeration and can act as templates for nanoparticles growth. So, the obtained results confirm the explanation maintained above for UV-visible spectroscopic analysis and the residual reducing sugars formed during the reaction.

Figure 7(a) shows TEM image of silver nanoparticles prepared with 1080 ppm silver nanoparticles (increasing the concentration of silver nitrate and starch was done while the ratio of silver nitrate to starch is maintained), which clarifies that although the concentration of silver nitrate is maximum, small nanosized spherical particles still exist and stabilized in colloidal and suspend form, which indicates that starch solution is still not saturated with silver nanoparticles as no agglomeration of nanoparticles takes place. Also the histogram clearly ensured that the prepared particle size lay in the range of 2–4 nm (50%) (Figure 7(b)). Silver nanoparticles solutions with such unique characteristics are unequivocally feasible for industrial applications.

## 5. Conclusion

Small sized spherical silver nanoparticles were successfully prepared by using alkali treated maize starch as reductant and stabilizing agent. The role of starch as a reducer comparing to other monosaccharides was monitored by measuring the redox potentials. The formation of spherical AgNPs was followed up, was controlled, and was firstly indicated by changing the color of solution to golden yellow giving absorbance peak around 400 nm. Secondly, the presence of spherical shaped AgNPs was confirmed by transmission electron microscope. The size distribution of spherical AgNPs was detected using transmission electron microscope photos and was in the range of 2–30 nm. At the optimum condition used, the average size of spherical AgNPs was 4–6 nm. The reduction process of Ag^+^ to Ag^0^ was followed up, controlled, and confirmed by measuring the reducing sugars in the reaction mixture using DNS reagent. This study reveals that a novel green method was used for synthesis of spherical AgNPs. The method used in the current work shows significant advantages, making it suitable for different industrial applications compared with the traditional methods for preparation of small sized spherical AgNPs, as it is a quite simple technique, with low chemical consumption and energy saving and no harazad organic solvents or reducing agents were used.

## References

[1] Hutchison J. E. (2008). Greener nanoscience: a proactive approach to advancing applications and reducing implications of nanotechnology. *ACS Nano*.

[2] Kamat P. V. (2002). Photophysical, photochemical and photocatalytic aspects of metal nanoparticles. *Journal of Physical Chemistry B*.

[3] Li L.-S., Hu J., Yang W., Alivisatos A. P. (2001). Band gap variation of size- and shape-controlled colloidal CdSe quantum rods. *Nano Letters*.

[4] Knoll B., Keilmann F. (1999). Near-field probing of vibrational absorption for chemical microscopy. *Nature*.

[5] Sengupta S., Eavarone D., Capila I. (2005). Temporal targeting of tumour cells and neovasculature with a nanoscale delivery system. *Nature*.

[6] Wiley B., Sun Y., Xia Y. (2007). Synthesis of silver nanostructures with controlled shapes and properties. *Accounts of Chemical Research*.

[7] Frattini A., Pellegri N., Nicastro D., de Sanctis O. (2005). Effect of amine groups in the synthesis of Ag nanoparticles using aminosilanes. *Materials Chemistry and Physics*.

[8] Tao A., Sinsermsuksakul P., Yang P. (2006). Polyhedral silver nanocrystals with distinct scattering signatures. *Angewandte Chemie (International Edition)*.

[9] Wiley B., Sun Y., Mayers B., Xia Y. (2005). Shape-controlled synthesis of metal nanostructures: the case of silver. *Chemistry—A European Journal*.

[10] Lisiecki I., Pileni M. P. (1993). Synthesis of copper metallic clusters using reverse micelles as microreactors. *Journal of the American Chemical Society*.

[11] Yanagihara N., Tanaka Y., Okamoto H. (2001). Formation of silver nanoparticles in poly(methyl methacrylate) by UV irradiation. *Chemistry Letters*.

[12] Kuo P. L., Chen W. F. (2003). Formation of silver nanoparticles under structured amino groups in pseudo-dendritic poly(allylamine) derivatives. *Journal of Physical Chemistry B*.

[13] Dong S. K., Lee T., Geckeler K. E. (2005). Hole-doped single-walled carbon nanotubes: ornamenting with gold nanoparticles in water. *Angewandte Chemie—International Edition*.

[14] Chen D.-H., Chen Y.-Y. (2002). Synthesis of strontium ferrite nanoparticles by coprecipitation in the presence of polyacrylic acid. *Materials Research Bulletin*.

[15] Qi L., Gao Y., Ma J. (1999). Synthesis of ribbons of silver nanoparticles in lamellar liquid crystals. *Colloids and Surfaces A: Physicochemical and Engineering Aspects*.

[16] Behrens S., Habicht W., Wagner K., Unger E. (2006). Assembly of nanoparticle ring structures based on protein templates. *Advanced Materials*.

[17] Naik R. R., Jones S. E., Murray C. J., McAuliffe J. C., Vaia R. A., Stone M. O. (2004). Peptide templates for nanoparticle synthesis derived from polymerase chain reaction-driven phage display. *Advanced Functional Materials*.

[18] Crooks R. M., Zhao M., Sun L., Chechik V., Yeung L. K. (2001). Dendrimer-encapsulated metal nanoparticles: synthesis, characterization, and applications to catalysis. *Accounts of Chemical Research*.

[19] Sun X., Jiang X., Dong S., Wang E. (2003). One-step synthesis and size control of dendrimer-protected gold nanoparticles: a heat-treatment-based strategy. *Macromolecular Rapid Communications*.

[20] Okugaichi A., Torigoe K., Yoshimura T., Esumi K. (2006). Interaction of cationic gold nanoparticles and carboxylate-terminated poly (amidoamine) dendrimers. *Colloids and Surfaces a: Physicochemical and Engineering Aspects*.

[21] Zhao M., Crooks R. M. (1999). Intradendrimer exchange of metal nanoparticles. *Chemistry of Materials*.

[22] Zhang J., Xu S., Kumacheva E. (2004). Polymer microgels: reactors for semiconductor, metal, and magnetic nanoparticles. *Journal of the American Chemical Society*.

[23] Geckeler K. E. (2002). *Advanced Macromolecular and Supramolecular Materials and Processes*.

[24] Shirtcliffe N., Nickel U., Schneider S. (1999). Reproducible preparation of silver sols with small particle size using borohydride reduction: for use as nuclei for preparation of larger particles. *Journal of Colloid and Interface Science*.

[25] Nickel U., Castell A. Z., Pöppl K., Schneider S. (2000). Silver colloid produced by reduction with hydrazine as support for highly sensitive surface-enhanced Raman spectroscopy. *Langmuir*.

[26] Chou K.-S., Ren C.-Y. (2000). Synthesis of nanosized silver particles by chemical reduction method. *Materials Chemistry and Physics*.

[27] Evanoff D. D., Chumanov G. (2004). Size-controlled synthesis of nanoparticles. II. Measurement of extinction, scattering, and absorption cross sections. *Journal of Physical Chemistry B*.

[28] Sondi I., Goia D. V., Matijević E. (2003). Preparation of highly concentrated stable dispersions of uniform silver nanoparticles. *Journal of Colloid and Interface Science*.

[29] Merga G., Wilson R., Lynn G., Milosavljevic B. H., Meisel D. (2007). Redox catalysis on “naked” silver nanoparticles. *Journal of Physical Chemistry C*.

[30] Ahmadi T. S., Wang Z. L., Green T. C., Henglein A., El-Sayed M. A. (1996). Shape-controlled synthesis of colloidal platinum nanoparticles. *Science*.

[31] Freeman R. G., Grabar K. C., Allison K. J. (1995). Self-assembled metal colloid monolayers: an approach to SERS substrates. *Science*.

[32] Ulman A. (1996). Formation and structure of self-assembled monolayers. *Chemical Reviews*.

[33] Zhao M., Sun L., Crooks R. M. (1998). Preparation of Cu nanoclusters within dendrimer templates. *Journal of the American Chemical Society*.

[34] Wang R., Yang J., Zheng Z., Carducci M. D., Jiao J., Seraphin S. (2001). Dendron-controlled nucleation and growth of gold nanoparticles. *Angewandte Chemie International Edition*.

[35] Zheng J., Stevenson M. S., Hikida R. S., Van Patten P. G. (2002). Influence of pH on dendrimer-protected nanoparticles. *Journal of Physical Chemistry B*.

[36] Petit C., Lixon P., Pileni M.-P. (1993). In situ synthesis of silver nanocluster in AOT reverse micelles. *Journal of Physical Chemistry*.

[37] El-Rafie M. H., El-Naggar M. E., Ramadan M. A., Fouda M. M. G., Al-Deyab S. S., Hebeish A. (2011). Environmental synthesis of silver nanoparticles using hydroxypropyl starch and their characterization. *Carbohydrate Polymers*.

[38] Hebeish A. A., El-Rafie M. H., Abdel-Mohdy F. A., Abdel-Halim E. S., Emam H. E. (2010). Carboxymethyl cellulose for green synthesis and stabilization of silver nanoparticles. *Carbohydrate Polymers*.

[39] Suslick K. S., Fang M., Hyeon T. (1996). Sonochemical synthesis of iron colloids. *Journal of the American Chemical Society*.

[40] Nam J.-M., Thaxton C. S., Mirkin C. A. (2003). Nanoparticle-based bio-bar codes for the ultrasensitive detection of proteins. *Science*.

[41] Schultz S., Smith D. R., Mock J. J., Schultz D. A. (2000). Single-target molecule detection with nonbleaching multicolor optical immunolabels. *Proceedings of the National Academy of Sciences of the United States of America*.

[42] Vasileva P., Donkova B., Karadjova I., Dushkin C. (2011). Synthesis of starch-stabilized silver nanoparticles and their application as a surface plasmon resonance-based sensor of hydrogen peroxide. *Colloids and Surfaces a: Physicochemical and Engineering Aspects*.

[43] Poliakoff M., Anastas P. (2001). Green chemistry: a principled stance. *Nature*.

[44] Sharma V. K., Yngard R. A., Lin Y. (2009). Silver nanoparticles: green synthesis and their antimicrobial activities. *Advances in Colloid and Interface Science*.

[45] Raveendran P., Fu J., Wallen S. L. (2003). Completely “Green” synthesis and stabilization of metal nanoparticles. *Journal of the American Chemical Society*.

[46] Raveendran P., Fu J., Wallen S. L. (2006). A simple and “green” method for the synthesis of Au, Ag, and Au-Ag alloy nanoparticles. *Green Chemistry*.

[47] Emam H. E., Manian A. P., Široká B. (2013). Treatments to impart antimicrobial activity to clothing and household cellulosic-textiles—why “nano”-silver?. *Journal of Cleaner Production*.

[48] Emam H. E., Mowafi S., Mashaly H. M., Rehan M. (2014). Production of antibacterial colored viscose fibers using in situ prepared spherical Ag nanoparticles. *Carbohydrate Polymers*.

[49] Huang H., Yuan Q., Yang X. (2004). Preparation and characterization of metal-chitosan nanocomposites. *Colloids and Surfaces B: Biointerfaces*.

[50] Abdel-Mohsen A. M., Aly A. S., Hrdina R., El-Aref A. T. (2012). A novel method for the preparation of silver/chitosan-O-methoxy polyethylene glycol core shell nanoparticles. *Journal of Polymers and the Environment*.

[51] Mohan Y. M., Raju K. M., Sambasivudu K., Singh S., Sreedhar B. (2007). Preparation of acacia-stabilized silver nanoparticles: a green approach. *Journal of Applied Polymer Science*.

[52] Keerthi Devi D., Veera Pratap S., Haritha R., Samba Sivudu K., Radhika P., Sreedhar B. (2011). Gum acacia as a facile reducing, stabilizing, and templating agent for palladium nanoparticles. *Journal of Applied Polymer Science*.

[53] Sreedhar B., Surendra Reddy P., Keerthi Devi D. (2009). Direct one-pot reductive amination of aldehydes with nitroarenes in a domino fashion: catalysis by gum-acacia-stabilized palladium nanoparticles. *Journal of Organic Chemistry*.

[54] Zahran M. K., Ahmed H. B., El-Rafie M. H. (2014). Alginate mediate for synthesis controllable sized AgNPs. *Carbohydrate Polymers*.

[55] Zahran M. K., Ahmed H. B., El-Rafie M. H. (2014). Facile size-regulated synthesis of silver nanoparticles using pectin. *Carbohydrate Polymers*.

[56] Vigneshwaran N., Nachane R. P., Balasubramanya R. H., Varadarajan P. V. (2006). A novel one-pot “green” synthesis of stable silver nanoparticles using soluble starch. *Carbohydrate Research*.

[57] Batabyal S. K., Basu C., Das A. R., Sanyal G. S. (2007). Green chemical synthesis of silver nanowires and microfibers using starch. *Journal of Biobased Materials and Bioenergy*.

[58] Brown W. H., Poon T. (2005). *Introduction to Organic Chemistry*.

[59] Gallant D. J., Bouchet B., Baldwin P. M. (1997). Microscopy of starch: Evidence of a new level of granule organization. *Carbohydrate Polymers*.

[60] Myers A. M., Morell M. K., James M. G., Ball S. G. (2000). Recent progress toward understanding biosynthesis of the amylopectin crystal. *Plant Physiology*.

[61] Sumner J. B. (1921). Dinitrosalicylic acid: a reagent for the estimation of sugar in normal and diabetic urine. *Journal of Biology and Chemistry*.

[62] Goia D. V. (2004). Preparation and formation mechanisms of uniform metallic particles in homogeneous solutions. *Journal of Materials Chemistry*.

[63] Blackburn R. S., Harvey A. (2004). Green chemistry methods in sulfur dyeing: application of various reducing D-sugars and analysis of the importance of optimum redox potential. *Environmental Science and Technology*.

[64] Miller G. L. (1959). Use of dinitrosalicylic acid reagent for determination of reducing sugar. *Analytical Chemistry*.

[65] Xu G.-N., Qiao X.-L., Qiu X.-L., Chen J.-G. (2008). Preparation and characterization of stable monodisperse silver nanoparticles via photoreduction. *Colloids and Surfaces A: Physicochemical and Engineering Aspects*.

